# MR and CT findings of cyst degeneration of sphenoid bone in McCune-Albright syndrome: a case report

**DOI:** 10.1186/1757-1626-2-9376

**Published:** 2009-12-22

**Authors:** Ping Li, Zai-Ren Zhang, Ying Jiang, Xu-Dong Xia, Dan Wang, Xian-Feng Li

**Affiliations:** 1 Department of Radiology, the Second Affiliated Hospital, Harbin Medical University, 246 Xue Fu Road, Harbin, Heilongjiang, 150086, China; 2 Department of Pathology, the Second Affiliated Hospital, Harbin Medical University, 246 Xue Fu Road, Harbin, Heilongjiang, 150086, China; 3 Department of Neurosurgery, the Second Affiliated Hospital, Harbin Medical University, 246 Xue Fu Road, Harbin, Heilongjiang, 150086, China

## Abstract

**Introduction:**

McCune-Albright syndrome (MAS) is a rare disorder characterized by the classic triad of precocious puberty, polyostotic fibrous dysplasia and café-au-lait pigmented skin lesions. Cystic change is rare in fibrous dysplasia (FD), especially in McCune-Albright syndrome. There were no reports about cyst degeneration in MAS which resulted in abnormal visual acuity and visual fields. Herein, we report a female patient with MAS associated with sphenoid bone cysts which resulted in visual deterioration to describe the computed tomography (CT) and magnetic resonance (MR) imaging findings of cyst degeneration in McCune-Albright syndrome.

**Case presentation:**

A 20-year-old female presented with right temporal hemianopsia and visual loss in the right eye suddenly. A café-au-lait spot was found on her neck and left shoulder. Endocrinologic examination revealed elevated basal level of serum PRL, FT_3 _and FT_4 _with decreased serum TSH. Fibrous dysplasia (FD) generally manifest as round-glass appearance with well defined borders and cystic areas within involved bone were seen as hypointensity on CT. They were showed as hypointense in T1-weighted sequences and as hyperintense in T2-weighted sequences of MRI. After surgery the right temporal hemianopsia improved.

**Conclusion:**

CT combined with MRI is the most effective method to evaluate the extent and complications of fibrous dysplasia in patients with MAS. The treatment of surgery can not cure MAS but relieve the symptom.

## Introduction

Fibrous dysplasia (FD) is primarily a developmental abnormality of the bone-forming mesenchyme in which fibrous tissue gradually expands and replaces the bone. It is a common benign bone disease existing in monostotic and polyostotic forms. Polyostotic fibrous dysplasia is a component of McCune-Albright syndromes (MAS). MAS is a rare disorder characterized by the classic triad of precocious puberty, polyostotic fibrous dysplasia and café-au-lait pigmented skin lesions. Additional endocrine abnormalities may also be present, including hyperthyroidism, growth hormone excess and hyperprolactinemia.

Cystic change is rare in fibrous dysplasia, especially in MAS [1]. There were no reports about cyst degeneration of FD in MAS which resulted in abnormal visual acuity and visual fields. Herein, we report a female patient with MAS associated with sphenoid bone cysts which resulted in visual deterioration.

## Case presentation

**Figure 1:**
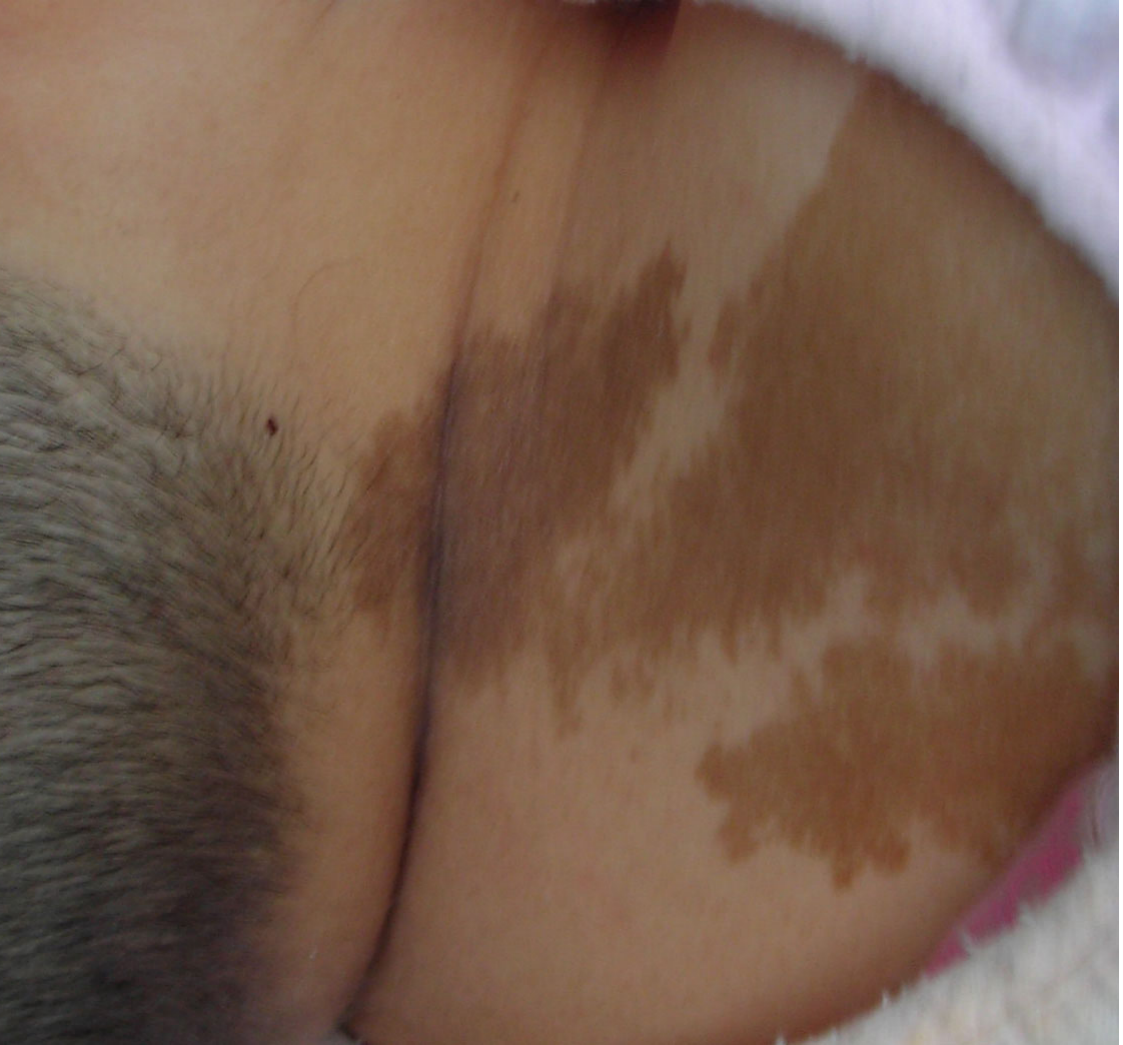
**A café-au-lait spot was found on her neck and left shoulder**.

A 20-year-old female presented with right temporal hemianopsia and visual loss in the right eye. She presented café-au-lait spots first noted at 2 months old. Her vision deteriorated suddenly when she was in hospital for ankle joint sprain 10 days ago. On admission, she had no acromegalic features, was 165 cm tall, weighing 66 kg. The blood pressure was 100/80 mmHg. The right side of his face was slightly deformed and proptosis of her right eyes was obvious. A café-au-lait spot was found on her neck and left shoulder (Fig. 1). The skin lesions were enlarged gradually during past 20 years. Endocrinologic examination revealed elevated basal level of serum PRL (687.29 nIU/ml, normal < 513 nIU/ml), FT_3 _(9.76 pmol/L, normal < 5.7 pmol/L) and FT_4 _(27.69 pmol/L, normal < 19.5 pmol/L) with decreased serum TSH (0.0011 μIU/ml, normal 0.35 μIU/ml ~4.94 μIU/ml). Other endocrine functions were normal.

**Figure 2:**
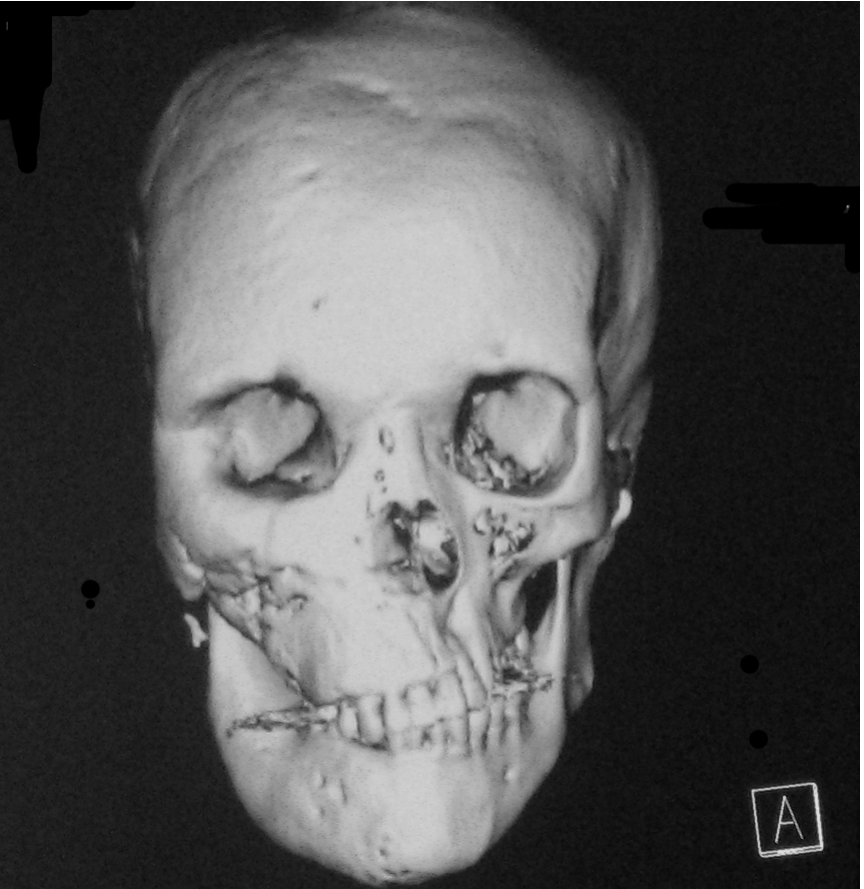
**Reconstructed three-dimensional CT image shows lion face appearance due to asymmetrical involvement and enlargement of the bones of the face and the skull**.

**Figure 3:**
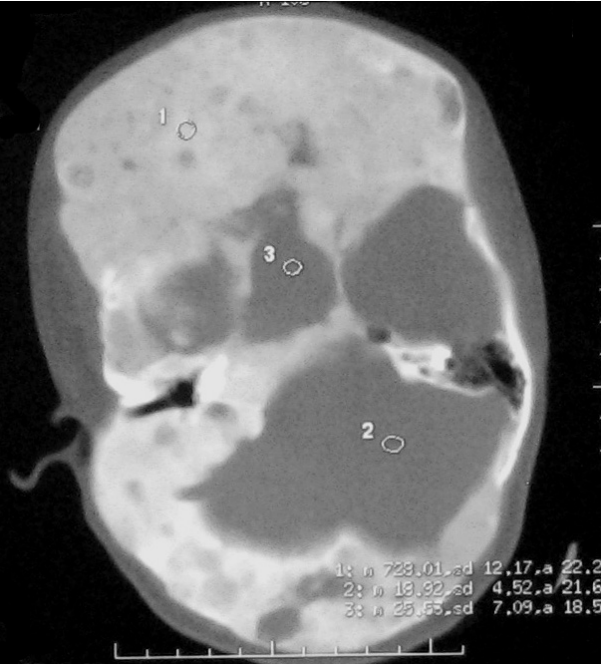
**Axial CT image FD presents as "ground-glass" appearance on CT and there is a cystic and solid mass of sella turcica**.

**Figure 4:**
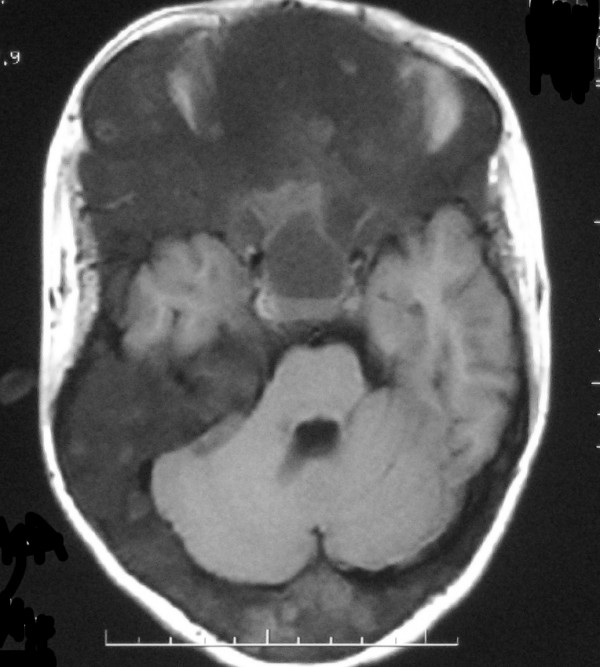
A**xial T1-weighted MR images show abnormal thickening of the bone involving the frontal bone, sphenoid bone, lateral and medial orbital walls, with right canal is shallow and optic nerve is compressed**.

**Figure 5:**
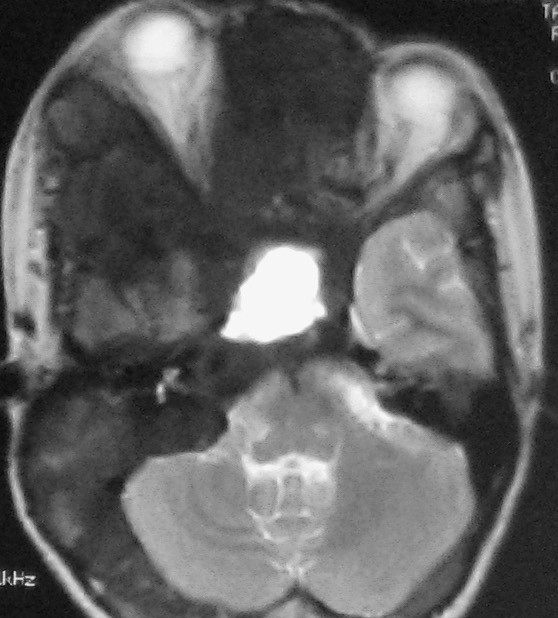
**Axial T2-weighted MR images show abnormal thickening of the bone involving the frontal bone, sphenoid bone, lateral and medial orbital walls, with right canal is shallow and optic nerve is compressed**.

**Figure 6:**
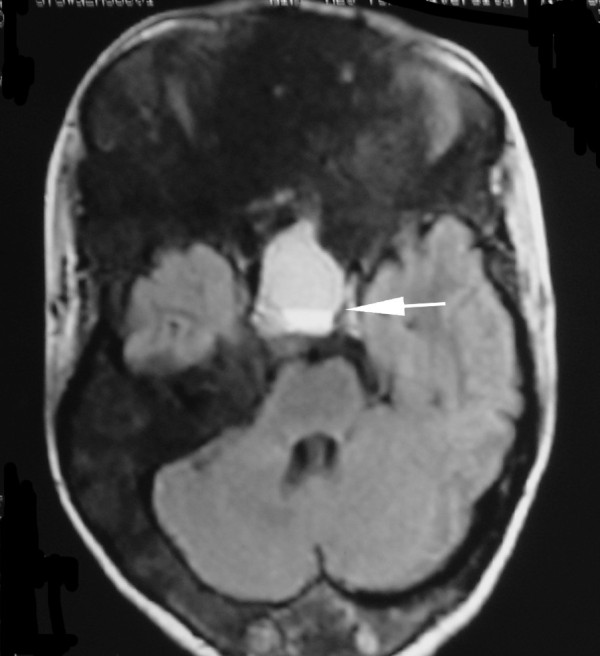
**Fluid-fluid level can be showed clearly on FLAIR T1-weighted images (arrow)**.

**Figure 7:**
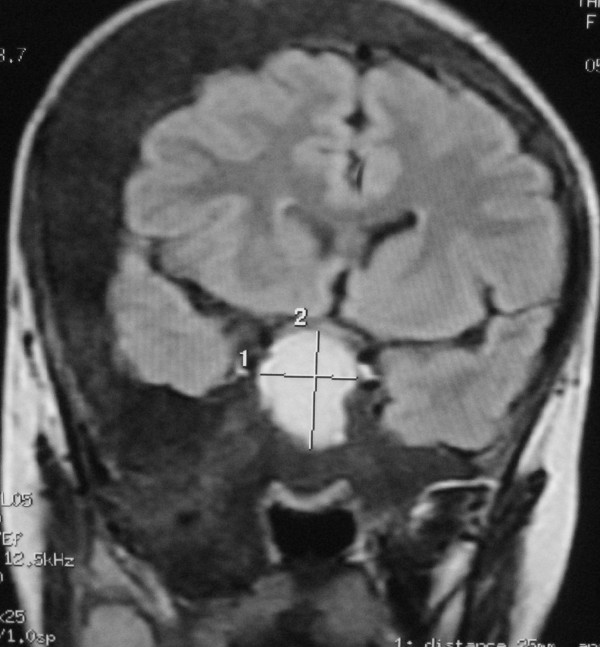
**T1-weighted sagittal MRI scans of the head**. Optic chiasma is compressed.

**Figure 8:**
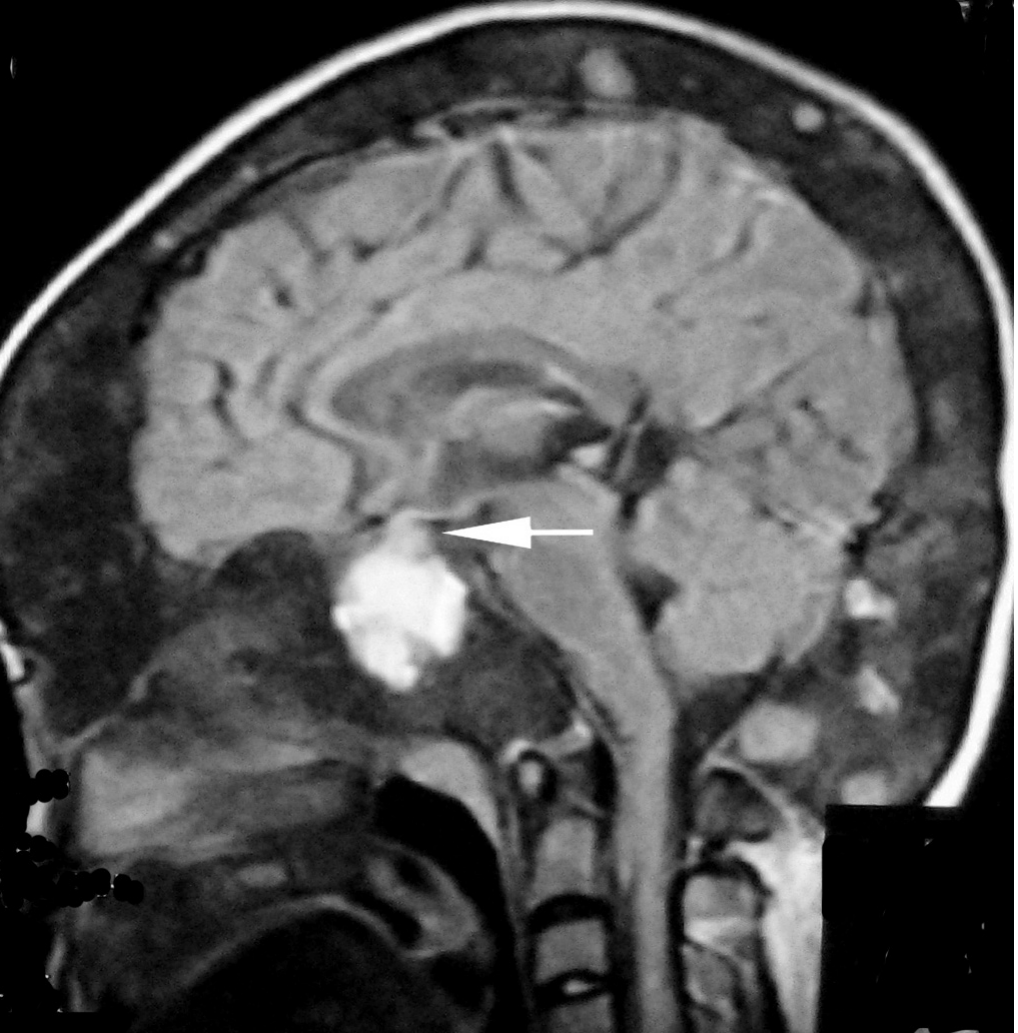
**T1-weighted coronal MRI scans of the head**. Normal pituitary gland can be found (arrow). The fluid-filled nature of the lesion is demonstrated by the fluid-fluid level.

**Figure 9:**
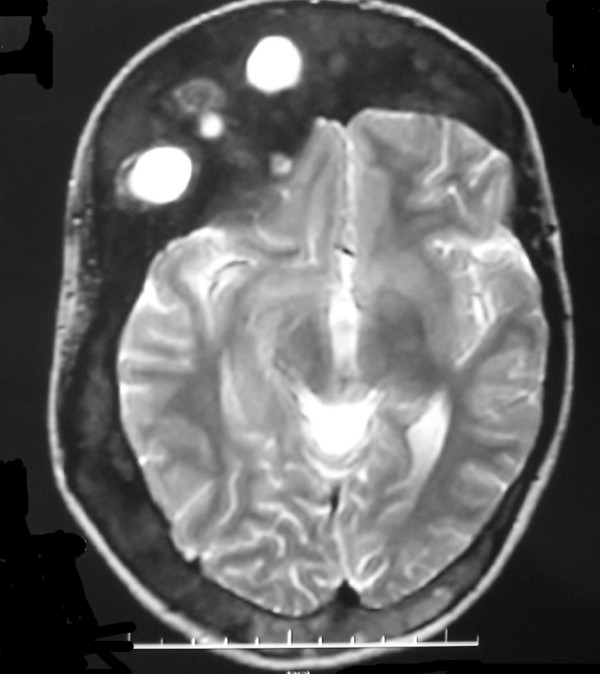
**T2-weighted MR image show cysts of frontal bone manifest as hypertensity with well-defined**. These lesions are more frequent in the craniofacial bones.

**Figure 10:**
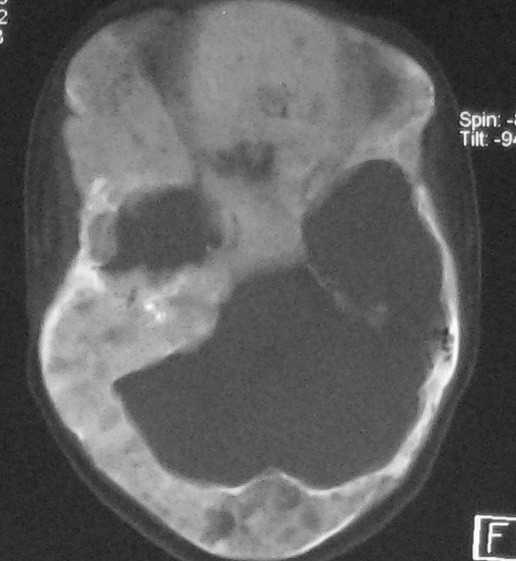
**Axial CT images**. There were no cyst changes in sphenoid bone 2 months ago. The density of sellar turcica is uniform.

CT and MRI scans of the head showed extensive abnormal thickening of the skull involving the right frontal, occipital, sphenoid and maxillary bones. Asymmetrical widening of facial bones resulted in lion face (leontiasis ossea) that is best showed with three-dimensional CT images (Fig. 2). Fibrous dysplasia caused narrowing of the optic canal and compression of the right optic nerve. Admission CT and MR scans revealed a cystic mass of sella turcica containing a fluid-fluid level (Fig. 3, 4, 5, 6). There was associated narrowing of the right optic canal and compression of the optic chiasma. On sagittal images of MR, normal pituitary gland can be found (Fig. 7, 8). There were several other cysts manifested as hypertensity on T2 weighted images in frontal bone (Fig. 9). Her visual acuity and visual fields were normal 2 months ago. There were no cyst changes in sphenoid bone at that time (Fig. 10).

**Figure 11:**
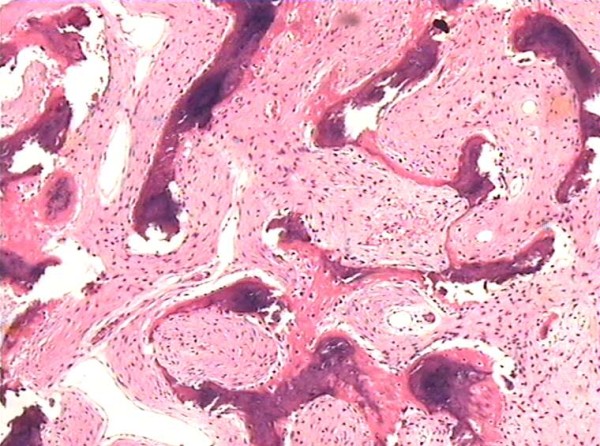
**The histology of fibrous dysplasia reveals fibroblasts, collagen, and scattered islands of cartilage, occasional giant cells and woven bone with excess blood supply**. HE ×400.

In view of the clinical presentation, cystic pituitary adenoma complicated with hemorrhage was suspected at first, but radiological appearances of the lesion showed that cyst degeneration of fibrous dysplasia can not be excluded because the normal pituitary gland can be seen. Surgery was performed by subfrontal approach. Right sphenoid bone was removed partially and the fluid in the cyst was drained. The fluid was lucid and there was no sign of hemorrhage. The walls of the cyst did not collapse. Tissues obtained at surgery was immediately fixed in 10% formalin and embedded in paraffin. Histological examination of the biopsied bone confirmed the presence of fibrous dysplasia. The histology of fibrous dysplasia reveals fibroblasts, collagen, and scattered islands of cartilage, occasional giant cells and woven bone with excess blood supply (Fig. 11). After surgery the right temporal hemianopsia improved.

## Discussion

FD is a developmental disease of bone in which there is replacement of normal spongiosa and filling of the medullary cavity of affected bones by an abnormal fibrous tissue that contains trabeculae of poorly calcified primitive bone formed by osseous metaplasia. It is believed to be a benign lesion of bone of unknown origin. Fibrous dysplasia might be monostotic or polyostotic, involve a large area of the skull or involve single bones. The most severe form of FD is McCune-Albright syndrome, which is more commonly found in females[2].

The McCune-Albright Syndrome is a sporadic rare disorder first described in 1936 by McCune and separately by Albright [3,4]. It is a rare disease with estimated prevalence between 1/100,000 and 1/1,000,000. McCune-Albright syndrome (MAS) is characterized by the association of precocious puberty (mostly in girls), polyostotic fibrous dysplasia and café-au-lait pigmented skin lesions. In addition to this classical triad, several endocrine disorders, all due to autonomous hormonal hyperproduction, can also be associated, such as pituitary adenomas secreting growth hormone, hyperthyroid goiters, or adrenal hyperplasia. The most commonly encountered endocrine dysfunction is gonadal hyperfunction. MAS is caused by a postzygotic activating mutation of the Gsalpha gene, which impair the guanosine triphosphatase (GTPase) activity of Gsalpha thus resulting in excess intracellular cAMP and leading to a mosaic distribution of cells bearing constitutively active adenyl cyclase activity[5].

Craniofacial structures are affected in 25%~30% of patients with fibrous dysplasia, most commonly involving the frontal bone, followed by the sphenoid and ethmoid bones. Facial bone involvement may cause visual disturbances, proptosis, nasalobstruction, sinusitis, facial asymmetry or hearing abnormalities. When the sphenoid ridge is involved, the commonest presenting sign is proptosis. Lee et al. concluded that encasement of the optic canal in fibrous dysplasia causes narrowing of the canal but this in itself does not result in visual loss [2,6]. Acute visual loss due to fibrous dysplasia is uncommon and in such chroinic cases optic nerve decompression does not lead to visual impairment. Cyst degeneration occurred most commonly in the sphenoid and frontal bones. Pressure on the optic nerves by a cyst is one of the causes of acute optic nerve compression in fibrous dysplasia [7]. The cysts tend to have a more aggressive course. It may be that the development of cyst degeneration does not depend on a single pathogenetic event, but that there are several potential mechanisms. Some cysts are thought to develop from disruption of venous diploic channels. It has also been proposed that bone cysts arise from an intraosseous vascular defect that results in intramedullary hemorrhage. The cysts can expand rapidly and produce symptoms which vary, depending on the location [8,9]. The development of cyst degeneration may cause rapid expansion which resulted in sudden visual deterioration. This 20-year-old female presented with right temporal hemianopsia and visual loss in the right eye quickly after the cyst developed.

Diagnosis is usually easy in polyostotic FD and MAS according to the imaging [10]. There were three patterns of the radiographic features of fibrous dysplasia of the skull and facial bones. The pagetoid or ground-glass pattern is most common and consists of a mixture of dense and radiolucent areas of fibrosis. This is due to the relatively greater degree of mineralization of FD tissue in the craniofacial bones [11]. Sclerotic lesions are homogeneously dense, whereas cyst degeneration is the least common finding is characterized by a spherical or ovoid lucency surrounded by a dense bony shell. Histologic correlates have shown cystic lesions result from an abundance of fibrous elements [8].

Computed tomography (CT) scanning is the "gold-standard" imaging technique for FD, allowing characterization of the three main imaging patterns of involved bone. On CT, FD generally presents as "ground-glass" appearance with well defined borders, medullary widening and cortical thinning. Cyst degeneration has occurred in about 5% of the patients with FD in the NIH cohort (unpublished data) [8]. Cystic areas within involved bone were seen as hypointensity on CT. CT should be repeated periodically, annually before stability has been demonstrated. MR imaging are useful adjuncts. Particularly in cases of cystic fibrous dysplasia, MR imaging may be useful to assess the soft tissue and fibrous components and to evaluate the effect of these primarily bony lesions on adjacent soft tissue structures of the skull base, especially sellar turcica. MRI can provide the exact delimitation of the lesions. Normal pituitary gland can be found on sagittal MR images in this case, so cystic pituitary adenoma can be excluded. FD manifest as intermediate or low signal intensity in T1-weighted MRI sequences, and intermediate or high signal intensity in T2-weighted MRI sequences. If fibrous tissue predominates, both T1- and T2-weighted signal intensity may be low [12]. Cystic fibrous dysplasia was showed as hypointense in T1-weighted images and as hyperintense in T2-weighted images of MRI. There was no heterogeneous contrast enhancement. A useful role for routine MR imaging of the sella turcica in all cases of clinically suspected McCune-Albright syndrome is suggested. While bone marrow involvement was shown more clearly with MRI, compression of cranial and spinal nerves was determined most effectively by evaluation of CT and MRI together. CT and MRI should be employed together in order to demonstrate the extent of disease, and complications of craniospinal involvement of FD in patients with MAS.

The treatment of MAS is complex and can vary from observation in asymptomatic cases to medical treatment or extensive surgery. The goal of the surgical treatment of MAS of the skull is to remove neural decompression of FD or mass. Total excision of the abnormal bone is not possible owing to its extent or involvement of the skull base, and treatment is limited to symptomatic relief.

## Conclusions

In conclusion, FD of skull generally manifest as round-glass appearance with well defined borders and cystic areas within involved bone were seen as hypointensity on CT. They were showed as hypointense in T1-weighted sequences and as hyperintense in T2-weighted sequences of MRI. CT combined with MRI is the most important method to evaluate the extent and complications of fibrous dysplasia in patients with MAS. The treatment of surgery can not cure MAS but relieve the symptom.

## Abbreviations

CT: Computed tomography; MR: magnetic resonance; MAS: McCune-Albright syndrome; FD: Fibrous dysplasia.

## Consent

Written informed consent was obtained from the patient for publication of this case report and accompanying images.

## Competing interests

The authors declare that they have no competing interests.

## Authors' contributions

All of the authors were involved in the clinical and radiographic assessment and writing the manuscript. All authors have read and approved the final manuscript.
